# The British Journals: Anatomy and Physiology

**Published:** 1839-10

**Authors:** 


### III. THE BRITISH JOURNALS.

(FOR THE QUARTER ENDING AUGUST 31, 1839.*)

### ANATOMY AND PHYSIOLOGY.

*On the Structure and Functions of the Spleen.* By THOMAS G. HAKE, M.D. *Communicated by* FRANCIS KIERNAN, Esq., F.R.S.

THE author, passing in review the various opinions which have been advanced by anatomists respecting the intimate structure of the spleen, arrives at the conclusion that hitherto only vague and premature inductions have been made. It is generally admitted that the fibrous envelope of this organ is formed of the external fibres of the splenic vein; and that from the internal surface of this envelope fibrous prolongations are continued into the interior of its substance, giving support to a fine cellular membrane, which is continuous with their edges, and variously reflected so as to constitute cells. The parenchyma, or solid structure of the spleen, everywhere accompanies these membranous productions, and forms the exterior walls of the cells, being composed of branches of the splenic arteries, of the granular terminations of those arteries constituting the *splenic grains* of Malpighi, of *venules*, which ramify around the splenic grains, and of *cellules*, into which the venules open, and from which the splenic veins take their rise. The author concludes, as the result of his enquiries, that a dilatable cellular tissue exists, containing venous blood, between the granules within which the arteries terminate, and the venules on the outer side of the splenic grains: that the venous membrane, which is continued from the cells to the cellules, as well as to the venules, becoming more and more attenuated, but without changing its essential structure, gradually loses its tubular form, and resumes its primitive character of cellular tissue; and that the artery, in like manner, is limited in its distribution within the granules by a cellular structure, which becomes vicarious of it, and determines the function it has to perform.

The author, in conclusion, offers some observations on the probable functions of the spleen. He considers the opinion which supposes that organ to be distended, at particular times, with arterial blood, as being completely refuted by the evidence derived from the preceding account of its minute structure; and suggests the probability of the spleen being rather a diverticulum for venous blood.

The paper is accompanied by seven highly-finished drawings, illustrating the structures described.

*Proceedings of the Royal Society. June* 20, 1839. No. 39.

---

*On the Difference of Colour in different parts of the Bodies of Animals.* By JAMES ALDERSON, M.A. M.D., late Fellow of Pembroke College, Cambridge. *Communicated by* P. M. ROGET, M.D., Sec. R.S.

THE hypothesis advanced by the author in explanation of the well-known partial absence of the coloured pigment or *rete mucosum,* in different parts of the human body, and that of other animals, is that it is due to the union or adhesion of the epidermis and the true skin, so as to exclude the rete mucosum. He supports this hypothesis by the analogy of a cicatrix, which is the result of an organization of a certain portion of lymph, poured out from the cut surfaces of a wound, as part of the process of nutrition, or as the consequence of a small amount of inflammation, induced either from mechanical irritation, or other accidental circumstance. This hypothesis was suggested by the colourless appearance of the cicatrix from the section of the umbilical cord in the negro, and also of that seen by the author at the umbilicus of the bottle-nosed whale, the *Hyperoodon bidentatus.* *Ibid.*

* Owing to the large amount of our other materials, we are forced greatly to contract this department of the Journal for the present quarter.—ED.

*On the Blood-vessels of Tendinous Tissues.* By James Paget, Demonstrator of Pathological Anatomy, and Curator of the Museum at St. Bartholomew's Hospital.

In this paper, which is very creditable to the author, Mr. Paget demonstrates the distribution of the larger vessels in tendons, which does not seem to have been previously effected; although, as Mr. P. observes, it does not appear why this has not been done, as he succeeded by using the common material for injection, size and vermilion. "The vessels in the substance of the tendon run in straight and parallel lines, from one end of the tendon to the other, between the fasciculi, rarely giving off branches and rarely anastomosing. Of the few branches which are given off, the greater number separate gradually and at a very acute angle from the trunk, and then pursue their course parallel to it; but occasionally a branch passes transversely across the intermediate tendinous fibres, from one vessel to another adjacent to it. The vessels of the substance communicate but rarely with those of the sheath of the tendon, and are derived from the vessels of the muscle or of the part in which the tendon expands to be inserted; so that I have sometimes succeeded in completely injecting both ends of a tendon, while the vessels of the middle portion remained empty. Each artery is accompanied by a single vein." *Lond. Med. Gazette. July*, 1839.

---

*On the Effects of Lesion of the Trunk of the Ganglionic System of Nerves in the Neck upon the Eye-ball and its appendages.* By John Reid, m.d., Fellow of the Royal College of Physicians, Edinburgh.

This is a brief paper, containing the details of several experiments on living animals, recently instituted with the view of determining a doubtful point, noticed in a previous publication. We have not room for the experiments, but the results obtained are stated in the preliminary observations, which we shall extract.

"In a former communication in the Edinburgh Journal (for January, 1838, p. 132,) I stated that I had frequently verified the observations of Petit and others, that when the vagus is injured in the neck in those animals in which the sympathetic is combined with the vagus, as in the dog,—that the conjunctiva becomes inflamed, the pupil contracts, and the eyelids are somewhat more closely approximated to each other. At that time I suggested that the contracted pupil and partially closed eyelids might probably depend upon the impatience of light which sometimes accompanies inflammation of the conjunctiva. I have since attended more carefully to this subject, and, during the last summer, satisfied myself that the contraction of the pupil, the projection of the cartilaginous membrane, or third eyelid, situated at the inner angle of the eye over the cornea, and the partial approximation of the eyelids to each other, take place immediately after the injury of the sympathetic, and before the inflammation of the conjunctiva presents itself, and that they continue after it has disappeared."

*Edin. Med. and Surg. Journal. July*, 1839.

